# Nonsurgical Management of Sinonasal Teratocarcinosarcoma With Chemoradiotherapy: A Case Report

**DOI:** 10.1155/crot/1109193

**Published:** 2025-10-09

**Authors:** Yuki Ban, Junko Tsuda, Yosuke Okinaka, Youhei Yamamoto, Mei Sakamoto, Tomoyasu Yamagata, Yosuke Takemoto, Makoto Hashimoto, Yoshinobu Hoshii, Kazuma Sugahara

**Affiliations:** ^1^Department of Otolaryngology, Yamaguchi University Graduate School of Medicine, Ube, Yamaguchi, Japan; ^2^Department of Diagnostic Pathology, Yamaguchi University Hospital, Ube, Yamaguchi, Japan

**Keywords:** chemoradiotherapy, immature squamous epithelium, immunohistochemistry, sinonasal teratocarcinosarcoma

## Abstract

Sinonasal teratocarcinosarcoma (SNTCS) is a rare, aggressive malignancy with epithelial, mesenchymal, and neuroectodermal components. We report the case of a 66-year-old man with right nasal obstruction and epistaxis. Imaging revealed a mass in the right nasal cavity and ethmoid sinus. Histopathology revealed immature squamous nests with clear cytoplasm, and immunohistochemistry confirmed multiphenotypic differentiation. Therefore, SNTCS was diagnosed. Owing to inoperability, the patient underwent chemoradiotherapy, which resulted in stable disease at 6 months. This case highlights the diagnostic complexity of SNTCS and suggests that nonsurgical management may be effective in select cases.

## 1. Introduction

Sinonasal teratocarcinosarcoma (SNTCS) is an extremely rare and aggressive malignancy that arises in the nasal cavity and paranasal sinuses. The average survival rate has been reported to be < 2 years, with a 3-year survival rate below 40%. Histologically, SNTCS consists of one or more epithelial components and multiple mesenchymal elements, demonstrating complex tissue heterogeneity. Owing to this diversity, diagnosis using small biopsy specimens is often challenging. Herein, we report a case of SNTCS arising in the paranasal sinus and provide a brief literature review.

## 2. Case Presentation

A 66-year-old man presented with right nasal obstruction and epistaxis. He first noticed these symptoms 3 months before visiting a local ENT clinic, where a bleeding nasal mass was identified. The patient was immediately referred to our hospital for further evaluation and treatment. He had no history of chronic sinusitis or notable family history. He was a current smoker who consumed 40 cigarettes per day.

### 2.1. Examinations

**Figure 1:**
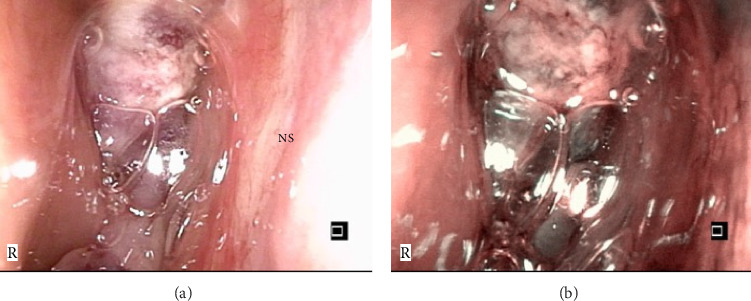
Initial nasal endoscopy findings. (a, b) Endoscopic views of the right nasal cavity. A dark reddish mass is observed anterior to the middle turbinate. No active bleeding is present. NS: nasal septum.

Nasal endoscopy revealed a dark reddish mass in the right nasal cavity anterior to the middle turbinate, with no active bleeding (Figure 1). The findings of the left nasal cavity were unremarkable. Blood tests showed a mild elevation in squamous cell carcinoma (SCC) antigen levels (2.6 ng/mL), while other tumor markers (NSE, ProGRP, and soluble IL-2 receptor) were within normal limits.

**Figure 2:**
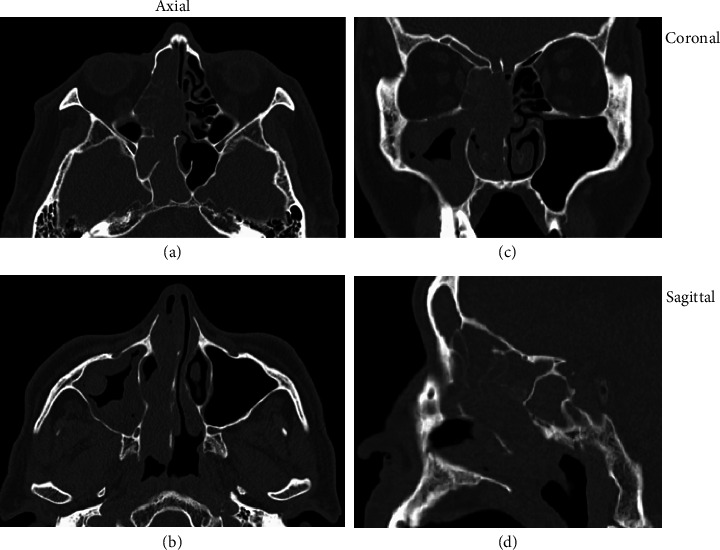
Computed tomography findings. (a, b) Axial CT images show a soft tissue mass occupying the right paranasal sinuses. Image (a) is taken at the level of the sphenoid sinus, whereas image (b) is taken at the level of the maxillary sinus. The nasal septum is deviated to the left. (c) Coronal CT view and (d) sagittal CT view show no evidence of bone destruction or orbital invasion.

**Figure 3:**
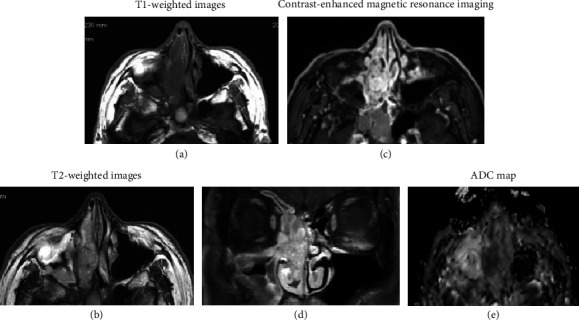
Magnetic resonance imaging findings. Axial (a) T1-weighted and (b) T2-weighted images show a mass with low signal intensity on T1 and mildly high signal intensity on T2 in the right paranasal sinus. (c) Contrast-enhanced T1-weighted image shows strong enhancement of the lesion. (d) Coronal Contrast-enhanced T1-weighted image demonstrates a hyperintense signal surrounding the mass. (e) Diffusion-weighted image shows high signal intensity in the periphery of the lesion, with a corresponding low apparent diffusion coefficient value, suggesting malignancy.

Computed tomography revealed a soft tissue mass filling the right paranasal sinuses, with mild deviation of the nasal septum to the left (Figure 2). No obvious bone erosion or orbital extension was observed. Magnetic resonance imaging revealed a mass in the right paranasal sinus with low signal intensity on T1-weighted images and mildly high signal intensity on T2-weighted images (Figures 3(a), 3(b)). The lesion exhibited strong contrast enhancement (Figures 3(c), 3(d)), and diffusion-weighted imaging demonstrated high peripheral signal intensity with a reduced apparent diffusion coefficient value (Figure 3(e)). The low apparent diffusion coefficient value indicated malignancy.

Histopathological examination of the biopsy specimen revealed granulation tissue with necrosis and infiltration of inflammatory cells. Although immunohistochemistry revealed partial positivity for MNF116, CK7, and p40, a definitive diagnosis was not established.

### 2.2. Treatment Course

Owing to inconclusive biopsy findings and radiological suspicion of malignancy, endoscopic tumor resection under general anesthesia was planned. On admission, the mass was significantly enlarged and protruded from both anterior and posterior nasal apertures. Endoscopic resection was performed using a navigation system. Intraoperatively, the tumor was debulked, and rapid pathology revealed small round cells suggestive of lymphoma or undifferentiated malignancy, which required further immunohistochemical analysis.

Additional tissue was resected from the superior and common nasal meatus, along with purulent drainage from the maxillary sinus. Lavage and decompression of the maxillary and ethmoid sinuses were then performed. Hemostasis was achieved, and the surgical cavity was packed.

**Figure 4:**
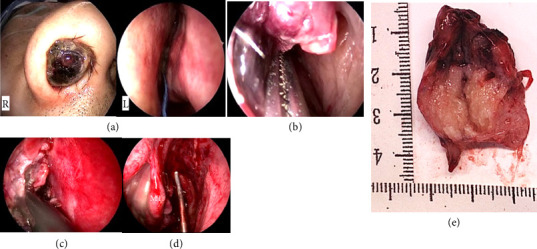
Intraoperative findings and resected specimen. (a) Preoperative view shows a large mass filling the right nasal cavity. (b) The tumor is reduced using a gauze soaked in cocaine and adrenaline to improve visualization. (c) The tumor appears to originate near the olfactory cleft. Biopsy specimens are obtained; rapid intraoperative diagnosis excludes epithelial carcinoma and malignant melanoma. (d) The mass is resected using a coblator, and a sufficient volume of tissue is collected. (e) Gross appearance of the resected specimen.

The patient had an uneventful postoperative course and was discharged on Postoperative Day 8. Intraoperatively, a large, friable tumor occupying the right nasal cavity was visualized. To facilitate manipulation, the tumor was reduced using a gauze soaked in cocaine and adrenaline. The tumor base appeared to originate near the olfactory cleft. Biopsy specimens were obtained, and rapid intraoperative diagnosis excluded epithelial carcinomas and malignant melanomas. The tumor was resected using a coblator, and a sufficient quantity of tissue was collected for histopathological analysis (Figure 4).

**Figure 5:**
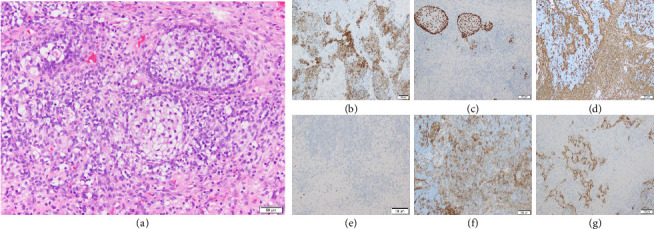
Histopathological findings and immunohistochemistry. (a) Hematoxylin and eosin staining reveal immature squamous nests with clear cytoplasm and intermixed poorly differentiated malignant cells. (b) MNF116 immunostaining is diffusely positive in epithelial components. (c) p40 shows nuclear positivity, supporting squamous differentiation. (d) Vimentin is strongly expressed in mesenchymal tumor components. (e) Myogenin is focally positive in rhabdomyoblastic elements. (f) CD99 is expressed in small round cells, consistent with primitive features. (g) S100 shows patchy positivity, indicating neuroectodermal differentiation.

The final pathological examination confirmed the diagnosis of SNTCS. Histological examination revealed immature squamous nests with clear cytoplasm and intermingled malignant components, exhibiting multiphenotypic differentiation (Figure 5).

Positron emission tomography–computed tomography revealed no residual tumor, lymph node involvement, or distant metastases. The patient declined additional surgery or carbon ion therapy because of the lack of residual disease and concerns about travel during the coronavirus disease 2019 pandemic. Definitive chemoradiotherapy was administered with cisplatin at a standard intended dose of 100 mg/m^2^ every 3 weeks. Owing to reduced creatinine clearance, the actual doses were reduced: 129.7 mg (Cycle 1, 80% dose), 126.4 mg (Cycle 2, 80% dose), and 78 mg (Cycle 3, 50% dose, because of reduced creatinine clearance and Grade 1 leukopenia). The cumulative cisplatin dose was 334.1 mg. Concurrent intensity-modulated radiotherapy (IMRT) was delivered at 2 Gy per fraction, 5 fractions per week, for a total of 60 Gy in 30 fractions. Six months after treatment, there was no evidence of recurrence.

## 3. Discussion

SNTCS is an extremely rare and aggressive malignant tumor that arises in the nasal cavity and paranasal sinuses. SNTCS was first described in the 1980s and is histologically characterized by the presence of malignant epithelial, mesenchymal, and neuroectodermal components [1, 2]. It demonstrates remarkable histological heterogeneity, including immature squamous epithelium, glandular structures, neuroepithelial rosettes, rhabdomyoblastic cells, and spindle-cell sarcomatous elements [1, 3].

Diagnosis of SNTCS is often difficult. Owing to its diverse histology, small biopsy samples are frequently misdiagnosed as undifferentiated carcinoma, sarcoma, or olfactory neuroblastoma [4, 5]. In the present case, clusters of immature squamous cells with clear cytoplasm [5] were identified, and immunohistochemical analysis showed positivity for epithelial markers (MNF116 and AE1/AE3) and for p40, which is specific for squamous differentiation. CD99 was also positive. In addition, the tumor showed positivity for the mesenchymal marker vimentin. Neural markers, including S100 (peripheral nerve), GFAP (glial), and CD56, were positive, as well as rhabdomyoblastic markers such as myogenin and MyoD1. These findings are consistent with those of previous reports and support the diagnosis of SNTCS [1, 4].

Recent molecular studies have identified recurrent inactivation of SMARCA4 and mutations in CTNNB1, suggesting that these tumors may be driven by alterations in chromatin remodeling and Wnt signaling pathways [6]. Although we did not perform genetic testing in this case, these findings may aid in future classification or targeted therapeutic approaches.

The prognosis for SNTCS remains poor, with a reported 3-year survival rate of < 40%, even after aggressive treatment [1]. A recent systematic review also reported that multimodal therapy—combining surgery, radiotherapy, and chemotherapy—was associated with improved overall survival compared with monotherapy approaches [7]. Early diagnosis and complete surgical resection are critical to improve outcomes, and adjuvant radiotherapy and/or chemotherapy are often recommended. In the present case, endoscopic tumor resection was performed, followed by concurrent chemoradiotherapy with cisplatin and intensity-modulated radiation therapy. No evidence of recurrence was observed 6 months after treatment.

Given its rarity and complex pathology, case reports, such as ours, are valuable for deepening our understanding of SNTCS. This case highlights the importance of adequate tissue sampling, comprehensive histopathological evaluation, and aggressive multimodal treatment to manage this challenging disease.
